# Health-related Quality of Life using the EQ-5D-5L: normative utility scores in a Dutch female population

**DOI:** 10.1007/s11136-022-03271-3

**Published:** 2022-10-20

**Authors:** Marloes E. Clarijs, Lindy M. Kregting, Nicolien T. van Ravesteyn, Linetta B. Koppert, Ida J. Korfage

**Affiliations:** 1 Department of Oncologic and Gastro-Intestinal Surgery, Academic Breast Cancer Center, Erasmus University Medical Center Cancer Institute, Dr. Molewaterplein 40, 3015 GD, Rotterdam, The Netherlands; 2 Department of Public Health, Erasmus MC, University Medical Center Rotterdam, Rotterdam, The Netherlands

**Keywords:** Utility, Health-related quality of life, EQ-5D-5L, Cost-effectiveness

## Abstract

**Purpose:**

Normative utility scores represent the health related quality of life of the general population, are of utmost importance in cost-effectiveness studies and should reflect relevant sexes and age groups. The aim of this study was to estimate EQ-5D-5L normative utility scores in a population of Dutch females, stratified by age, and to compare these scores to those of female populations of three other countries.

**Methods:**

Dutch women completed the EQ-5D-5L online between January and July 2020. Mean normative utilities were computed using the Dutch EQ-5D-5L value set, stratified by age, tested for differences using the Kruskall–Wallis test, and compared to normative utility scores of female populations elsewhere. Additionally, to support the use of the Dutch EQ-5D-5L data in other settings, normative utility scores were also calculated by applying the value sets of Germany, United Kingdom and USA.

**Results:**

Data of 9037 women were analyzed and the weighted mean utility score was 0.911 (SD 0.155, 95% CI 0.908–0.914). The mean normative utility scores differed between age groups, showing lower scores in older females. Compared to other normative utility scores of female populations, Dutch mean utilities were consistently higher except for age groups 18–24 and 25–34. With the three country-specific value sets, new age-specific mean normative utility scores were provided.

**Conclusion:**

This study provides mean normative utility scores of a large cohort of Dutch females per age group, which were found to be lower in older age groups. Utility scores calculated with three other value sets were made available.

**Supplementary Information:**

The online version contains supplementary material available at 10.1007/s11136-022-03271-3.

## Plain English Summary

Health-related quality of life is a measure of the impact of disease and treatment on an individuals’ disability and daily functioning. Health-related quality of life outcomes are gathered using questionnaires (e.g. EQ-5D-5L) and respondents’ answers can be converted into a single utility score, that reflects an individual’s health state at a particular point in time. These utility scores are used in cost-effectiveness studies. Utilities that are obtained in the general population, instead of patients with a specific disease, are called normative utilities. Differences in normative utility scores between countries, age groups and gender have been found and choosing the most accurate set of normative utility scores is important. However, Dutch age and gender-specific normative utility scores for females are currently not available. This study converted the EQ-5D-5L results of 9037 women into mean normative utility scores stratified by age. Relatively high mean normative utility scores for the EQ-5D-5L in Dutch females were found in all age groups compared to female populations of other countries, with the lowest scores in older women. The EQ-5D-5L normative utility scores calculated with Dutch data and the value sets of Germany, United Kingdom and USA in this study support the use of the Dutch data in international cost-effectiveness studies when age and country-specific normative utility scores for women are not available.

## Introduction

The effectiveness of a health care intervention or strategy can be measured in a variety of ways. A commonly used method is measuring and comparing the Health-related Quality of Life (HrQoL) between groups. HrQoL is a measure of the impact of disease and treatment on an individuals’ disability and daily functioning [1]. It includes factors that are part of an individual’s health, without non-health aspects such as economic circumstances, and is often used in cost-effectiveness studies [2]. HrQoL outcomes are gathered using questionnaires and respondents’ answers can be converted into a single utility score, usually between 0 and 1, that reflects the personal desirability of an individual’s health state at a particular point in time [2]. The EQ-5D-5L is often recommended as the instrument to obtain utility scores [3]. To enable the conversion for EQ-5D-5L outcomes, pre-defined country-specific value sets have been developed to this aim [4].

In cost-effectiveness studies, utility scores are used to calculate quality adjusted life years (QALY’s) for all relevant health states. If utility scores are not available for these health states, assumptions about such utilities have to be made. However, assumptions are sub-optimal compared to objectively measured utilities as this influences cost-effectiveness ratios and ultimately decision-making [5, 6]. Besides utilities for disease specific health states, also utilities for the general population are considered to be relevant. These so-called ‘normative utility scores’ can be used as a comparator for health profiles of patients based on subgroups with similar age and gender. Additionally, they can be used to compensate for a loss in HrQoL due to factors that are not caused by the disease or intervention of interest [7]. Currently, many cost-effectiveness studies made the assumption of a utility of 1 (reflecting perfect health) for the general population. However, Versteegh et al. obtained utilities in a general Dutch population and the results suggested that utilities of the general population tend to be below one [8]. This means that cost-effectiveness studies may overestimate the health of the general population, and thereby overestimate the loss in utility score caused by a disease or intervention. Therefore, up to date normative utility scores are needed to be used in cost-effectiveness studies.

Other countries have calculated normative utility scores using the EQ-5D and showed differences between genders [9–11]. In studies on women’s health, using gender-specific normative EQ-5D utility scores of females only may be more accurate than population norms. Janssen et al. published EQ-5D index value population norms for 20 countries in Europe including the Netherlands [12, 13]. Data of 2367 people, identified between 2001 and 2003, were used to calculate age stratified normative utility scores [14]. However, these results were based on the EQ-5D-3L, and the Dutch normative data for the EQ-5D-5L that was published thereafter, were not classified by gender [8, 13]. This is a drawback for cost-effectiveness studies among only male or female populations.

Therefore, the aim of this study was to obtain EQ-5D-5L normative utility scores in a female Dutch cohort, stratified by age. In addition, these normative utility scores were compared to normative utility scores of female cohorts of other countries. Furthermore, three different country-specific value sets were applied to the answers of the EQ-5D-5L of the Dutch cohort. This analysis was conducted to illustrate the impact of using different value sets on age-specific mean normative utility scores, and to enable the use in cost-effectiveness studies in populations for which country-specific normative utility scores for women are not available.

## Methods

### Study participants

Data were collected in a study that initially obtained normative data for the Breast-Q (a breast cancer specific quality of life questionnaire) (Oemrawsingh et al. (2021), in press). Dutch women were invited to complete a web-based survey that was disseminated through social media platforms of the Erasmus Medical Center between January and July 2020. Because the researchers focused on breast cancer, normative data should be based on women unencumbered by the diagnosis of breast cancer. Therefore, women who were previously diagnosed with breast cancer were excluded from the survey.

Besides the Breast-Q, the survey also included the EQ-5D-5L. The current study made use of this EQ-5D-5L data.

### Health related quality of life measured with the EQ-5D-5L

The Dutch version of the EQ-5D-5L was used to measure HrQoL [3]. The EQ-5D-5L is a non-disease-specific instrument, and consists of five dimensions (mobility, self-care, usual activities, pain/discomfort and anxiety/depression), each with five levels of functioning, ranging from no problems to extreme problems. Eventually, 3125 different health states can be provided based on these five dimensions. A quality-adjustment weight or “utility” is a number anchored at 0 and 1, with “perfect health” carrying a weight of 1 and death carrying a weight of 0. A utility score below 0 is possible when a health state is valued worse than death. Utilities can be calculated after application of pre-defined values to a specific health state as indicated by a respondent. Utilities in this study were computed according to the Dutch tariffs for the EQ-5D-5L as established by Versteegh et al. [8].

### Statistical analysis

**Fig. 1:**
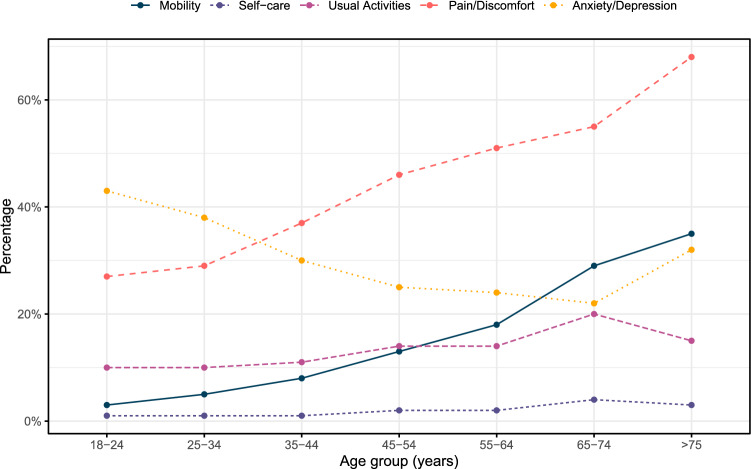
Frequencies of having “any problems” (level 2–5) in the EQ-5D-5L dimensions based on age group

Descriptive statistics, including standard deviations and confidence intervals, were calculated to present the mean normative EQ-5D-5L index scores per age group. Age was categorized into seven subgroups; 18–24, 25–34, 35–44, 45–54, 55–64, 65–74 and ≥ 75 years. A weighted mean normative utility score was calculated taking into account the population size per age group of the Dutch population in 2020 (See Online Appendix, Fig. 1) [15]. Because the data were not normally distributed, the Kruskal–Wallis test was used to compare mean utility scores between all age groups. The data analyses were performed using IBM SPSS Statistics (Version 25) and R (Version 1.2).

### Comparisons with three other countries

The mean normative utility scores per age group were compared to normative utility scores for female populations in studies performed in Germany, South Australia, and the USA (US) [9–11]. Furthermore, the country-specific value sets used in these studies (i.e. the value sets of Germany, the United Kingdom (UK) and the US) were also applied to the EQ-5D-5L data to convert them into utility scores [16–18].

## Results

The total sample included 9037 females with a median age of 46.0 years (range 18–90 years). According to the responses of the individual EQ-5D-5L dimensions, most health problems were identified in the pain/discomfort (41.2%) and anxiety/depression (29.5%) dimension (Table 1). The anxiety/depression dimension showed relatively high percentages of any health problems (level 2–5) in the younger age groups, which decreased with increasing age. Health problems in the other dimensions increased when becoming older, which was most evident in the mobility dimension (Fig. 1). The mean utility score was 0.917 (SD 0.110, 95% CI 0.915–0.920) with a left-skewed distribution, as 44.7% had a utility score of 1 (*n =* 4037). The weighted mean utility score was 0.911 (SD 0.155, 95% CI 0.908–0.914).

**Table 1:** Prevalence of EQ-5D-5L responses for the Dutch female normative population (*N =* 9037), stratified by age group

Level	Mobility		Self-care		Usual Activities		Pain/Discomfort		Anxiety/Depression	
	*N*	(%)	*N*	(%)	*N*	(%)	*N*	(%)	*N*	(%)
All ages										
1	8016	88.7	8899	98.5	7900	87.4	5328	59.0	6379	70.6
2	719	8.0	109	1.2	836	9.3	2871	31.8	2174	24.1
3	241	2.7	24	0.3	237	2.6	701	7.8	413	4.6
4	51	0.6	3	0.0	60	0.7	123	1.4	63	0.7
5	10	0.1	2	0.0	4	0.0	14	0.2	8	0.1
Any problems		*11.4*		*1.5*		*11.9*		*41.2*		*29.5*
Age 18–24										
1	614	96.8	628	99.1	573	90.4	460	72,6	361	56.9
2	15	2.4	6	0.9	52	8.2	146	23.0	203	32.0
3	4	0.6	0	0.0	7	1.1	25	3.9	58	9.1
4	1	0.2	0	0.0	2	0.3	2	0.3	9	1.4
5	0	0.0	0	0.0	0	0.0	1	0.2	3	0.5
Any problems		*3.2*		*0.9*		*9.6*		*27.4*		*43*
Age 25–34										
1	1498	95.5	1557	99.2	1419	90.4	1112	70.9	981	62.5
2	47	3.0	7	0.4	114	7.3	368	23.5	470	30.0
3	19	1.2	4	0.3	26	1.7	72	4.6	96	6.1
4	4	0.3	0	0.0	10	0.6	16	1.0	20	1.3
5	1	0.1	1	0.1	0	0.0	1	0.1	2	0.1
Any problems		*4.6*		*0.8*		*9.6*		*29.2*		*37.5*
Age 35–44										
1	1902	91,7	2048	98.7	1841	88.8	1306	63.0	1430	68.9
2	124	6,0	23	1.1	173	8.3	611	29.5	545	26.3
3	39	1,9	3	0.1	47	2.3	133	6.4	88	4.2
4	7	0,3	0	0.0	13	0.6	22	1.1	10	0.5
5	2	0,1	0	0.0	0	0.0	2	0.1	1	0.0
Any problems		*8.3*		*1.2*		*11.2*		*37.1*		*30.5*
Age 45–54										
1	2416	87.3	2715	98.1	2348	86.2	1488	53.8	2083	75.3
2	258	9.3	42	1.5	277	10.0	976	35.3	577	20.9
3	70	2.5	8	0.3	81	2.9	246	8.9	94	3.4
4	18	0.7	1	0.0	23	0.8	48	1.7	12	0.4
5	5	0.2	1	0.0	2	0.1	9	0.3	1	0.0
Any problems		*12.7*		*1.8*		*13.8*		*46.2*		*24.7*
Age 55–64										
1	1285	82.0	1541	98.3	1342	85.6	774	49.4	1194	76.1
2	199	12.7	21	1.3	162	10.3	603	38.5	302	19.3
3	70	4.5	6	0.4	54	3.4	166	10.6	62	4.0
4	12	0.8	0	0.0	9	0.6	24	1.5	9	0.6
5	2	0.1	0	0.0	1	0.1	1	0.1	1	0.1
Any problems		*18.1*		*1.7*		*14.4*		*50.7*		*24.0*
Age 65–74										
1	279	71.4	377	96.4	312	79.8	177	45.3	307	78.5
2	68	17.4	9	2.3	55	14.1	152	38.9	68	17.4
3	35	9.0	3	0.8	20	5.1	51	13.0	13	3.3
4	9	2.3	2	0.5	3	0.8	11	2.8	3	0.8
5	0	0.0	0	0.0	1	0.3	0	0.0	0	0.0
Any problems		*28.7*		*3.6*		*20.3*		*54.7*		*21.5*
Age > 75										
1	22	64.7	33	97.1	29	85.3	11	32.4	23	67.6
2	8	23.5	1	2.9	3	8.8	15	44.1	9	26.5
3	4	11.8	0	0.0	2	5.9	8	23.5	2	5.9
4	0	0.0	0	0.0	0	0.0	0	0.0	0	0.0
5	0	0.0	0	0.0	0	0.0	0	0.0	0	0.0
Any problems		*35.3*		*2.9*		*14.7*		*67.6*		*32.4*

EQ-5D-5L answer levels—level 1 (no problems), level 2 (slight problems), level 3 (moderate problems), level 4 (severe problems), level 5 (inability/extreme problems). Any problems—percentage of any problems (level 2–5) in the EQ-5D-5L dimensions according to age group

### Primary outcome

The mean normative utility score ranged from 0.929 (SD 0.102) (age group 25–34) to 0.881 (SD 0.081) (age group > 75). The highest mean normative utility scores were found in the three youngest age groups (between age 18 and 44 years) (Table 2). After age 45, mean normative utilities decreased with increasing age with lowest mean utility scores in the oldest age group (> 75 years). The Kruskal–Wallis test revealed that there were statistically significant differences in mean normative utility scores between all age groups (*p <* 0.001). However, absolute differences were small.

**Table 2:** Mean utility scores, standard deviations and confidence intervals of four different utility value sets applied on the Dutch female normative EQ-5D-5L data (*N =* 9037)

Age group	*N*	Dutch value set Versteegh et al. [8]		German value set Ludwig et al. [18]		UK value set Devlin et al. [17]		US value set Pickard et al. [16]	
		Mean (SD)	95% CI	Mean (SD)	95% CI	Mean (SD)	95% CI	Mean (SD)	95% CI
18–24	634	.927 (.091)	0.920–0.934	.953 (.075)	0.947–0.959	.933 (.083)	0.926–0.939	.934 (.094)	0.926–0.941
25–34	1569	.929 (.102)	0.924–0.934	.953 (.087)	0.949–0.957	.935 (.094)	0.930–0.940	.935 (.110)	0.929–0.940
35–44	2074	.925 (.102)	0.921–0.930	.950 (.087)	0.946–0.954	.933 (.095)	0.929–0.937	.931 (.114)	0.926–0.936
45–54	2767	.913 (.120)	0.908–0.917	.939 (.106)	0.935–0.943	.925 (.106)	0.921–0.929	.918 (.131)	0.913–0.923
55–64	1568	.907 (.112)	0.902–0.913	.936 (.098)	0.931–0.941	.919 (.102)	0.914–0.925	.910 (.127)	0.904–0.916
65–74	391	.890 (.131)	0.877–0.903	.919 (.117)	0.859–0.888	.901 (.123)	0.889–0.914	.884 (.154)	0.869–0.900
> 75	34	.881 (.081)	0.854–0.910	.918 (.066)	0.895–0.941	.894 (.084)	0.864–0.923	.877 (.108)	0.839–0.915

*N* number of participants per age group, *SD* standard deviation, *CI* confidence interval. *UK* United Kingdom, *US* USA

### Comparisons with three other countries

Compared to published normative utility scores for female populations in Germany, the US and South Australia, our mean normative utilities were consistently higher except for age groups 18–24 and 25–34 (Table 3).

**Table 3:** Mean normative utility scores based on the EQ-5D-5L in other female populations stratified by age group

Age group	The Netherlands		Germany Grochtdreis et al. [11]		South Australia McCaffrey et al. [10]		US Jiang et al. [9]	
	*N*	Mean (SD)	*N*	Mean (SD)	*N*	Mean (SD)	*N*	Mean (SD)
18–24	634	0.93 (0.09)	230	0.94 (0.08)	226	0.95 (0.08)	53	0.93 (0.09)
25–34	1569	0.93 (0.10)	363	0.92 (0.10)	224	0.95 (0.11)	130	0.92 (0.11)
35–44	2074	0.93 (0.10)	386	0.88 (0.17)	241	0.91 (0.13)	95	0.85 (0.21)
45–54	2767	0.91 (0.12)	494	0.86 (0.19)	253	0.87 (0.16)	102	0.81 (0.24)
55–64	1568	0.91 (0.11)	399	0.86 (0.20)	226	0.88 (0.15)	67	0.83 (0.21)
65–74	391	0.89 (0.13)	346	0.85 (0.25)	193	0.87 (0.16)	57	0.82 (0.22)
> 75	34	0.88 (0.08)	366	0.77 (0.31)	122	0.82 (0.15)	61	0.83 (0.18)
Total	9037	0.92 (0.11)	2584	0.86 (0.20)	1486	0.90 (0.14)	565	0.86 (0.19)

*N* number of participants per age-group, *SD* standard deviation, *SE* standard error

The mean utility scores were recalculated after applying the country-specific value sets of Germany, the UK, and the US to the EQ-5D-5L answers of our Dutch cohort. This resulted in slightly higher mean utility scores for all age groups with all three value sets (Table 2). The mean utility scores were the highest when the German value set was applied.

## Discussion

We obtained normative utility scores using the EQ-5D-5L in a sample of 9037 Dutch females and found relatively high utility values for Dutch females aged 18 to > 75 years old. In general, the mean normative utilities were lower in the older age groups although absolute differences were small. Applying the country-specific value sets of Germany, UK and US to the EQ-5D-5L answers of our Dutch sample resulted in consistently higher mean utility scores in all age groups as compared to the mean utility scores calculated with the Dutch value set.

Our mean normative utility scores in the younger age groups were slightly lower than previously found in female populations of other countries [9–11]. This difference may be caused by the sampling method. Young people that are less healthy may spend more time on their computer, mobile phones or social media than healthy adolescents who are possibly able to do more activities. Therefore, they might have been more likely to encounter the study invitation and more inclined to complete a questionnaire on their health. The normative utility data of female populations of other countries was collected between 2013 and 2017 [9–11]. The lower Dutch utilities in the younger age groups compared to those of previous studies might be explained by an increase in mental health problems in adolescents over the last years as observed in the Netherlands [19]. The data of this study were collected during the start of the COVID-19 pandemic, which also led to more anxiety and mental health issues particularly in females and adolescents, and may have contributed to lower utility scores [20]. Besides, it appears as if the use of the Dutch value set is partially responsible for the differences in utility scores in younger age groups (up to 35 years), because the differences in utility becomes smaller when the German, UK, and US value sets were used. In contrast, our mean normative utility scores in the older age groups were higher than those in female populations of other countries. Particular in these age groups, the differences were enlarged by the use of the German, UK and US value sets. That is, these differences cannot be explained by the value sets themselves.

The oldest age group (> 75 years) showed a relatively high mean normative utility, as none of the participants scored level four and five across all dimensions. This might indicate that older Dutch women have a relatively good quality of life, and possibly better than older women elsewhere. In contrast to a recently published Russian article reporting normative utility scores, Dutch women did not show many problems in the self-care dimension for all age groups [21]. In the current study, the frequency of having any problems in the anxiety/depression dimension decreased with increasing age, but was consistent across all age groups in the Russian population. Although the pattern of having any problems in the mobility dimension was similar in both studies, the frequency in the older age group was considerably higher in the Russian population [21]. However, the high mean normative utilities may also be related to most participants being between 75 and 80 years of age, and no one being older than 90 years. Because more health issues appear with increasing age, this may explain the differences with other studies if they included older participants [21–23]. In addition, the sample of older participants (*n =* 34) was relatively small, which reduces the generalizability. Another explanation is the use of social media as a recruitment method, which may have caused some selection bias. Older females that are able and willing to complete a questionnaire through an online survey are potentially in better health [24]. On the other hand, internet is easily accessible in the Netherlands and internet use is higher than in most other western countries, also in older people [25]. Interestingly, Jiang et al. has shown differences in outcome between face-to-face and online sampling, with higher EQ-5D-5L index scores in the face-to-face population for most age groups [9]. However, the index scores of the older participants (i.e. above the age of 65) were slightly higher in the online population [9].

We found statistically significant differences in mean normative utility scores between the age groups. However, we expected larger age-specific absolute differences beforehand based on results of previous normative studies (both males and females) in the Netherlands [26]. Nevertheless, we recommend to use age and gender-specific reference values, as they are important for cost-effectiveness studies and can have a substantial effect on outcomes [5, 6]. It would be interesting to investigate to what extent our age-specific values alter the outcomes of cost-effectiveness analyses. To note, our normative utility scores are mainly intended to answer women-specific research questions, and they might not be directly comparable to future normative utility scores of Dutch males as they are not generated from the same sample.

The key strengths of our study are the use of the EQ-5D-5L to obtain normative utility scores and the large sample size. The EQ-5D-5L is more sensitive than the EQ-5D-3L version which has several limitations (e.g. ceiling effects in patient populations, non-detection of small differences or changes in patients with mild conditions) [27–29]. Furthermore, the sample size of our cohort was substantially larger (at least three times) than the samples in previous studies, and in combination with the more sensitive 5-level version of the EQ-5D, our study may have resulted in more reliable outcomes [9–12]. Another strength is that we provide age-specific mean utility scores specifically for women. These could be used an up-to-date reference point in research and Dutch health policy evaluations, such as breast and cervical cancer screening strategies, and health policies for pregnancy and childbirth. Importantly, our study did not gather demographic data which makes it difficult to state anything about the representativeness of the population. We used a web-based survey that was disseminated through the institutes’ social media platforms, which are all accessible for the general population. To be able to complete the survey, access to internet was required. Especially in the Netherlands, internet use has increased over the last decade and is nowadays extremely high as 95% of total population has access to internet [30]. This makes the internet-user population very similar to the general population. Even back in 2013, internet was the main source to search for health information (83%) in the Netherlands, and social media is frequently used for this purpose [31]. The percentage of social media use is more than 90% for the age group of 18–54 years, and between 76 and 89% in the age group of 55–64 years of the Dutch population [32]. Although we cannot assume that all female internet-users have seen our survey, we believe that the survey reached a large and representative part of the Dutch female population. Despite our large sample size the group of elderly females was relatively small. In other countries where internet availability is less developed, using this sampling method might be more of an issue because certain populations are possibly left out.

To date, it is unclear if and to which extent utility measurements on a national level can be generalized to other countries. However, there are differences between the country-specific value sets even between countries that were expected to have quite similar populations, socioeconomic status, health systems, or attitudes to health [13]. Therefore, using a country-specific value set is encouraged [33, 34]. In this study, a subset of value sets of three other countries was used to calculate utility scores based on the answers to the EQ-5D-5L of our Dutch female cohort. This was done to illustrate the impact of using different value sets on age-specific mean normative utility scores, and also to provide age-specific mean normative utility scores to be used in cost-effectiveness studies in countries of which country-specific normative utility scores for women are lacking. For example, if a breast cancer study would be conducted in the UK, researchers probably prefer to use the UK value set to determine the utilities in patients. In order to allow for proper comparisons with the general population, they can also best use normative utilities calculated with the UK value set. If age-specific mean normative utility scores for women in the UK are not available, the normative utility scores calculated with the UK value set in this study may be a good alternative. Reporting the normative utility scores for different value sets enlarges the applicability in multiple international studies.

## Conclusions

In this study, we presented age-specific normative utility scores for the EQ-5D-5L in Dutch females using different value sets. We found lower mean normative utilities in older age groups. Relatively high normative utility scores were found in all age groups, compared to those in other female populations. Furthermore, utility scores were calculated with value sets of three other countries which can be used as normative comparisons in international patient populations.

## Supplementary Information

Below is the link to the electronic supplementary material.

Supplementary file1 (PDF 413 kb)

## Data Availability

The data are not publicly available but are available for researchers that wish to apply their own country-specific EQ-5D-5L value set on the current dataset. Data shall only be shared with researchers upon reasonably request, at the discretion of the principal investigator.
