# Supplementary material for: Health-related Quality of Life using the EQ-5D-5L: normative utility scores in a Dutch female population
Source: Qual Life Res. 2022 Oct 20;32(2):373–81. doi: 10.1007/s11136-022-03271-3 (PMC9584237; doi:10.1007/s11136-022-03271-3)

Appendix 1

Figure 1. Age group distribution for this current cohort (n = 9037) and the female Dutch population in 2020 (n = 7.1 million) used for the weighted mean normative utility score calculation.

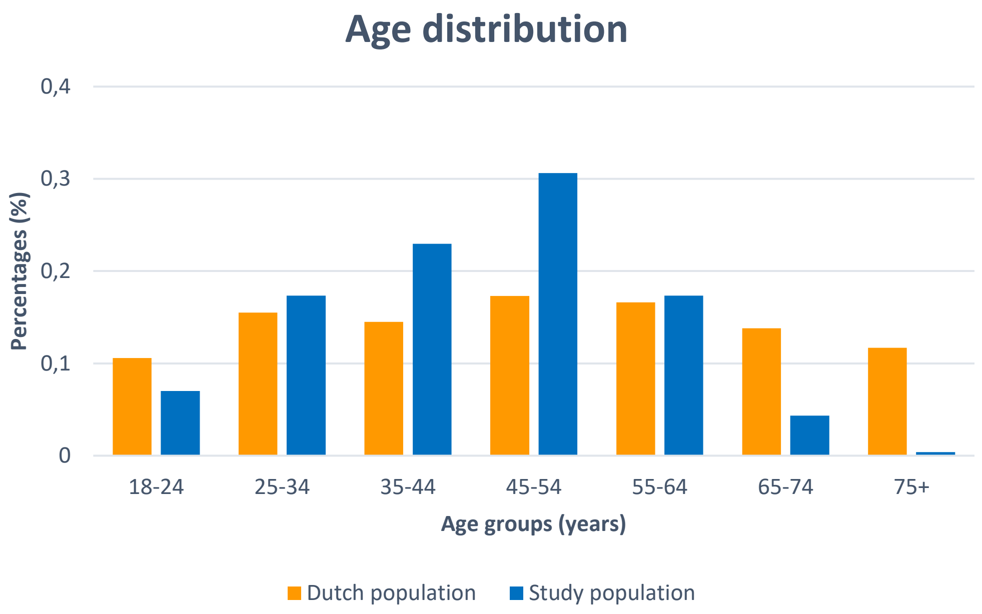

Supplement: Supplementary file 1 — Supplementary file1 (PDF 413 kb) [file 11136_2022_3271_MOESM1_ESM.pdf]
